# Predominantly Superior Retinal Tears Detected by B-Scan Ultrasonography

**DOI:** 10.1155/2019/7105246

**Published:** 2019-06-11

**Authors:** M. Streho, F. Perrenoud, N. Abraham, K. Hawa, M. Puech, A. Giocanti-Aurégan

**Affiliations:** ^1^Centre Explore Vision, Paris, France; ^2^Centre d'Exploration de la Vision, Rueil-Malmaison, France; ^3^Ophthalmology department, Lariboisière Hospital, APHP, Paris VII University, DHU Vision and Handicaps, Paris, France; ^4^Ophthalmology department, Avicenne Hospital, APHP, Paris XIII University, DHU Vision and Handicaps, Paris, France

## Abstract

**Introduction:**

When fundus examination is not possible, ultrasonography (US) is an accurate tool for the diagnosis of retinal tears (RT). The aim of this study was to describe the predominant location of RT, the factors influencing their location, and the vitreous status of eyes with RT using US.

**Methods:**

A prospective study was conducted in all patients diagnosed with RT by B-scan US (Aviso, Quantel Médical, Clermont-Ferrand, France). The primary endpoint was to assess RT preferential location using US. Secondary endpoints were the rate of posterior vitreous detachment (PVD), number of eyes with multiple RT, and axial length (AL).

**Results:**

A total of 101 eyes of 100 patients with RT were included. RT main location was in the superior quadrants (either nasal superior, strictly superior, or temporal superior) in 71% of cases. All patients were diagnosed with PVD by US, and 79% had a vitreous hemorrhage. Twelve eyes (13%) were diagnosed with multiple RT. The mean AL was 24.62 ± 2 mm, and it was significantly longer in eyes without superior RT (25.52 mm versus 24.37 mm; *p*=0.004).

**Conclusion:**

In this study, we showed a superior location of RT diagnosed by US in more than two-thirds of cases associated with a significantly shorter AL than in other locations. This finding could increase US sensitivity for RT detection and help to improve the US learning curve of ophthalmologists in training and surgical decision-making when the retina is inaccessible due to opacity media.

## 1. Introduction

Retinal tears are the main complications of an atypical detachment of the vitreous from the peripheral retinal surface [1]. Retinal tears are usually located in the anterior part of the posterior vitreous detachment (PVD) and have been reported in 8–22% of eyes with PVD [2–5]. The pathophysiology of retinal tear formation is suspected to be associated with the presence of a pathological PVD. However, the exact chronology is poorly understood.

Ultrasonography (US) is a very useful tool allowing an accurate examination of the retina when fundus examination cannot be performed due to opacity media, a situation that is more common in case of PVD associated with vitreous hemorrhage preventing to correctly examine the retina.

The aim of this study was to describe the predominant location of retinal tears, the factors influencing their location and the vitreous status of eyes with retinal tears using US.

## 2. Patients and Methods

**Figure 1:**
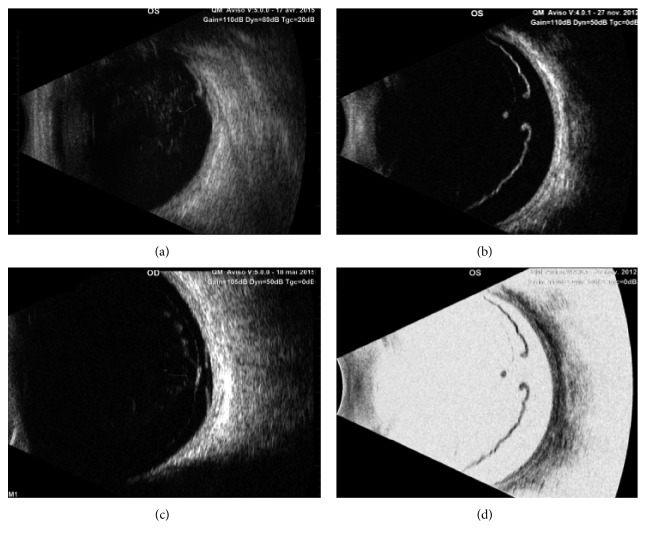
B-scan ultrasonography: (a) Eleven o'clock meridian acquired with a 10 MHz probe (Aviso, Quantel Médical) showing a typical retinal tear on B-scan ultrasonography. (b) Superior longitudinal scan acquired with a 20 MHz probe showing a typical retinal tear on B-scan ultrasonography. (c) Superior longitudinal scan acquired with a 20 MHz probe showing a retinal tear associated with retinal detachment. (d) Same pictures than in C with reversed grey scale for a better visualization of the retina and underlying tissue.

A prospective study was conducted in a tertiary center specialized in imaging and treatment of retinal diseases. All consecutive patients diagnosed with retinal tears by B-scan US between September 2015 and April 2016 were included in this study. All patients underwent B-scan US (Aviso, Quantel Médical, Clermont-Ferrand, France) performed by an experienced examiner with a 10 MHz probe and completed with a 20 MHz probe at the examiner discretion (Figure 1). This study was conducted in accordance with the tenets of the Declaration of Helsinki, and an informed consent was obtained from patients. Approval was obtained from the France Macula Federation ethics committee.

Inclusion criteria were as follows: all consecutive patients referred to the center for vitreous hemorrhage, retinal detachment, and PVD and diagnosed by B-scan US with retinal tear. Patients referred for laser treatment were also included when retinal tear was clinically diagnosed by B-scan US. Both eyes of the same patient could be included.

Exclusion criteria were as follows: patients referred for vitreous hemorrhage, retinal detachment, and PVD without retinal tear after US examination. Patients with tractional retinal detachment were also excluded.

All patients underwent a complete B-scan US examination performed by one of the 3 US specialists of the center (MS, MP, and FP) on which the diagnosis of retinal tear was based.

For this study, data collected were as follows: location of the retinal tear following the classification presented below, axial length (measured by US using the 10 MHz probe), vitreous status (PVD), number of retinal tears, and presence of vitreous adherences. PVD was classified as complete or incomplete and as involving the posterior pole or periphery. We considered an absence of PVD, when the posterior hyaloid was not seen either in static and dynamic examination, in every single field, incomplete PVD, when posterior hyaloid was partially seen, and complete PVD, when the visualization of posterior hyaloid was complete in every single field, including the posterior pole. Furthermore, traction effects of PVD on retinal tear flaps were assessed (as opposed to a fully operulated flap with no traction on the edge of the tear).

The examination technique was adapted from a method previously described by Lorenzo-Carrero et al. [1]. Patients were placed in a prone position on an examination table. Examination was performed with the 10 MHz probe on the eyelids. Then following topical anesthesia, the 20 MHz probe was positioned on the ocular surface through 2.5% methylcellulose when needed. The dB gain was adjusted when using each probe to provide the finest images of ocular structures. Kinetic examination was performed by assessing vitreous movements during voluntary eye movements, while the probe was kept immobile according to the previously described method [1].

All retinal tear locations were classified as follows: strictly superior, nasal superior, temporal superior, strictly nasal, nasal inferior, strictly inferior, temporal inferior, and strictly inferior or central.

The primary endpoint was to determine the preferential location of retinal tears detected by B-scan US.

Secondary endpoints were the rate of PVD, number of eyes with multiple retinal tears, and axial length.

Statistical analysis was performed using a *t*-test and a Fisher's exact test with Prism7 software. A *p*-value <0.05 was considered statistically significant.

## 3. Results

One-hundred and one eyes of 100 patients with retinal tears diagnosed on B-scan US examination were included. There were 53 men and 47 women with a mean age of 65 ± 10 years. Seventy-one percent of eyes were phakic, and 29% were pseudophakic. All patients (100%) were diagnosed with PVD on B-scan US, and 79% (80 eyes) had a vitreous hemorrhage. The PVD was partial in 78 eyes and complete in 23 eyes with both posterior pole and peripheral PVD.

**Figure 2:**
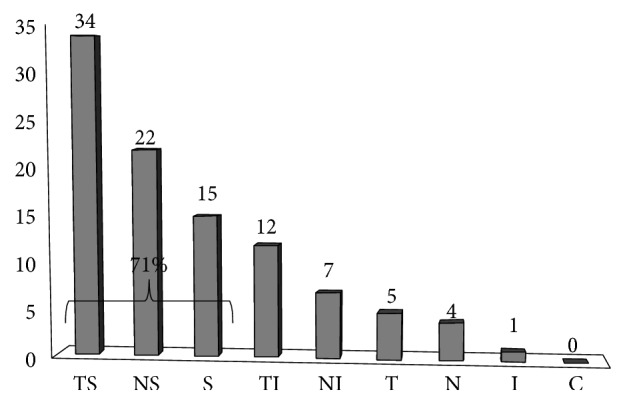
Retinal tear distribution on the retina (percentage of retinal tears per quadrant): strictly superior (S), nasal-superior (NS), temporal superior (TS), strictly nasal (N), nasal inferior (NI), strictly inferior (I), temporal inferior (TI), strictly inferior (I), or central (C).

The main location of the retinal tear was in the superior quadrants (either nasal superior, strictly superior, or temporal superior) in 71% of eyes and in another location in 29% of eyes. There was no difference of tear location between phakic and pseudophakic, respectively, 72% and 70.7% of superior tears.  Figure 2 presents the distribution of the different locations of retinal tears in our population following the classification presented in the method section. Twelve eyes (13%) were diagnosed with multiple tears, and the mean number of tears per eye was 2.5 (2–6).

Among eyes with vitreous hemorrhage (80 eyes), 65 (83.33%) were diagnosed with at least 1 superior tear (either nasal superior, strictly superior, or temporal superior), while 15 eyes did not show any tear in the superior quadrants (16.67%), but had tear in another location. Among eyes without vitreous hemorrhage (21 eyes), 13 (65.22%) were diagnosed with at least 1 superior tear (either nasal superior, strictly superior, or temporal superior), while 8 eyes did not show any tear in the superior quadrants (34.78%), but had tear in another location. The difference in prevalence of superior tears between eyes with and without vitreous hemorrhage was not significant (*p*=0.08).

A persistent vitreous traction on the flap was observed in 58 eyes (57.4%), the edges of the retinal tear were not flat in 33 eyes (32.7%), and in 7 eyes (6.9%), a retinal detachment was associated without any preferential retinal tear location.

**Figure 3:**
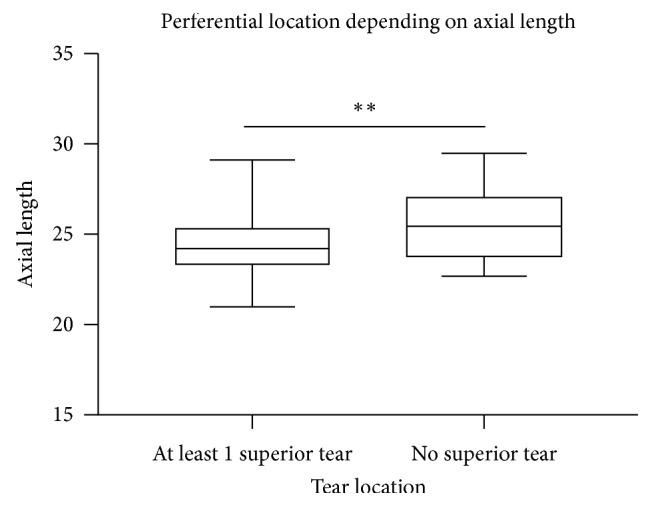
Comparison of the axial length between eyes with at least one superior retinal tear (*n* = 78) and eyes without any superior retinal tear (*n* = 23); *p*=0.004 (unpaired *t*-test).

The mean axial length was 24.62 ± 2 mm. Among the 78 eyes (Figure 3) with at least 1 superior retinal tear (either nasal superior, strictly superior, or temporal superior), the mean axial length was 24.37 ± 1.06 mm (21.0–29.1), while among the 23 eyes without any superior tear (either strictly nasal, nasal inferior, strictly inferior, temporal inferior, or strictly inferior), the mean axial length was 25.5 ± 1.4 mm (22.7–29.5), and the difference was statistically significant (*p*=0.004).

## 4. Discussion

In this prospective study based on B-scan US, we identified that the diagnosed retinal tears were located in the superior quadrants in more than two-thirds of cases.

The predominantly superior location of retinal tears identified based on the US examination seems consistent with the clinical findings described by Lincoff and Gieser [6] when investigating the presence of retinal hole in case of retinal detachment. However, it should be noted that, in our study, the rate of intravitreal hemorrhages was high (79%), and this could be due to the preferentially superior location of retinal tears and be symptomatic because of the effect of gravity on vitreous blood. This high rate of intravitreal hemorrhages could be due to our patient recruitment in our tertiary center since patients were referred for US examination, especially when the diagnosis of retinal tears could not have been made clinically when the fundus was not accessible for examination. This possible bias was ruled out by the fact that among patients with intravitreal hemorrhages, 83.33% had a superior location and a similar proportion (71%) was found in the overall population of patients in this study (*p*=0.08).

Clinical examination with a 3-mirror contact lens is the gold standard for the diagnosis of peripheral retinal tears in case of PVD. However, in case of retinal hemorrhage with no biomicroscopic access to the fundus, the decision of vitreous surgery is mainly based on US imaging. Therefore, it is essential to improve our knowledge of the preferential location of retinal tears, so that the examination may be mainly focused on the superior retina, with a more sensitive probe of 20 MHZ. Although the risk that a retinal tear may be missed by a trained examiner on B-scan US is very low given the high negative predictive value (99%) found in the literature [1], it is always important to improve the precision and the knowledge of this examination. In case of short-time examination required for a non-cooperative patient for instance, it is important to be rapidly efficient. In our series, in order to minimize the missed tears, we performed in our very specialized ultrasound center a systematic examination for all eyes: the 9 fields were systematically examined with a 10 MHz probe with maximal gain of 110 dB, and if there were any doubts, the examination was completed by a 20 MHz probe. Multiple tears were systematically sought. Another recent study confirmed a sensitivity of 100% for the detection of rhegmatogenous retinal detachment and for the detection of new retinal tears in patients without retinal detachment [7] using a standardized scanning protocol and a dedicated ophthalmic ultrasonographer, as it was the case in our study.

Moreover, in this study, we identified a possible orientation factor in the location detection of retinal tears, a shorter mean axial length being associated with locations in the superior quadrants, and a longer mean axial length with other locations (*p*=0.004). To the best of our knowledge, this is the first report of the association between a preferential location of retinal tears depending on the axial length in the literature. However, an association between the distance to the limbus and the axial length has already been suggested [8]. In our study, only 23 eyes presented without superior retinal tears, and larger studies are thus needed to confirm this association.

Knowing the preferential location of retinal tears could have several advantages: (i) it could increase the B-scan US sensitivity for detecting retinal tears, (ii) it could help to improve the B-scan US learning curve of ophthalmologists in training, and (iii) it could help to improve US examination reliability for surgery decision-making.

In this study, we also observed multiple retinal tears in 13% of cases. This proportion is high and motivates to continue the examination even once one retinal tear has been found.

In the present study, a fine adhesion of the vitreous to the edge of the retinal flap was found in 58 eyes (57.4%). This high proportion supports the commonly admitted hypothesis that retinal tears are caused by an excessive traction of the vitreous cortex on the peripheral retina, particularly in areas with a strong vitreous adhesion [9] probably increased by eye movements. For the remaining patients without adhesion on the retinal flap, the retinal tear could have been caused by the complete peripheral PVD at the time of the detachment.

Our study has some limitations: (i) the tertiary center recruitment could have selected a different population with more intravitreal hemorrhages with fundus often inaccessible for examination, (ii) no data were available on patient evolution after the diagnosis was made because our center is responsible for imaging exploration and laser treatment, but the surgical cases were referred to retinal surgeons of another department, and (iii) the number of patients is limited by the fact that we preferred to conduct a prospective analysis to limit biases.

In conclusion, we showed that, when diagnosed by B-scan US, retinal tears are located in the superior quadrants in more than two-thirds of cases. This finding could help US practices to more rapidly identify retinal tears by starting the examination in this area when a retinal tear is suspected and improve the US learning curve of ophthalmologists in training. Moreover, we suggest here an association between a shorter axial length and the presence of a superior retinal tear that should be confirmed in larger studies.

## Data Availability

All data are available as an excel document in the supplementary materials.

## Supplementary Materials

Supplementary Materials The supplementary file includes the data that support the findings of this study.
